# Immunochemical detection of glycated lens crystallins and their circulating autoantibodies in human serum during aging

**Published:** 2008-11-17

**Authors:** Mala Ranjan, Sujatha Nayak, Tanuja Kosuri, Beedu Sashidhar Rao

**Affiliations:** Department of Biochemistry, University College of Science, Osmania University, Hyderabad, India

## Abstract

**Purpose:**

The aim of this investigation was to exploit lens-specific glycated crystallins as an immunogen to detect human glycated crystallins and their circulating autoantibodies in human serum during aging in relation to the development of cataract.

**Methods:**

Polyclonal antibodies were produced against human total lens proteins (40–80 years) in rabbits. The specificity of the antibodies produced were determined by antibody capture assay using purified human lens crystallins (high molecular weight fraction [HMW]+α, HMW+α-glycated, β, β-glycated, γ, and γ-glycated) as antigens. The cross-reactivity of these lens specific antibodies against rat β-, β-glycated, γ-, and γ-glycated lens crystallins was also analyzed. A non-competitive enzyme linked immunosorbent assay (ELISA) methodology was developed for the detection of circulating lens crystallins in human sera using HMW+α, HMW+α-glycated, β-, and β-glycated crystallins from humans and γ- and γ-glycated crystallins from rats as immobilized antigens. Circulating autoantibodies were also detected in human sera by antibody capture assay. The methodology was validated by evaluating 60 human serum samples collected from cataract patients and 30 human serum samples from apparently normal subjects belonging to the same age group.

**Results:**

The polyclonal antibodies raised against human total lens proteins showed 90% and 65% cross-reactivity with rat γ- and β-crystallins, respectively, by ELISA. Further, these polyclonal antibodies were capable of detecting both native and in vitro synthesized glycated crystallins. Their IC_50_ values were observed to be (i) human total lens proteins (55 ng), (ii) human HMW+α (16.45 ng), (iii) human HMW+α-glycated (273 ng), (iv) human β- (37.82 ng), (v) human β-glycated (260 ng), (vi) rat γ- (105.34 ng), and (vii) rat γ-glycated (313 ng). The immunochemical analysis of human serum indicated a significant change (p<0.001) in the levels of circulating β-glycated and γ-glycated crystallins in the age group of 40–80 years with respect to their control groups. However, there was no statistically significant change in the levels of HMW+α-glycated crystallins in the age group of 40–80 years as compared to their age-matched controls. Notably, the levels of serum γ-glycated crystallins were found to be threefold higher than that of HMW+α-glycated and β-glycated crystallins in the age group of 70–80 years. Circulating autoantibodies to HMW+α-glycated, β-glycated, and γ-glycated crystallins were detected in the serum of both apparently normal and cataract patients in the age group of 40–80 years by antibody capture assay. The levels of these autoantibodies were significantly higher at every time point compared to their respective controls. Autoantibodies to γ-glycated crystallins were found to be twofold and 3.2 fold higher as compared to the levels of autoantibodies to β-glycated and HMW+α-glycated crystallins, respectively. Western blot and immunohistochemical analysis substantiated the observations made in non-competitive ELISA.

**Conclusions:**

During the course of aging, leakage of lens crystallins (HMW+α, HMW+α-glycated, β, β-glycated, γ, and γ-glycated) elicit an immune response resulting in the formation of autoantibodies in cataract patients (40–80 years) as compared to age matched controls. This is the first experimental report where polyclonal antibodies raised against lens-specific glycated crystallins were capable of detecting the early leakage of glycated crystallins in human subjects. This immunochemical approach has implications in the early detection of senile cataract.

## Introduction

Cataract includes any opacity of the lens from minor opacities not interfering with vision to total opacity causing blindness. Cataract is also classified as congenital, infantile, and age-related (senile). Senile cataract remains a major cause of blindness, affecting over 20 million of nearly 45 million blind people worldwide with the highest incidence occurring in developing countries [1-3]. There are no drugs available to treat cataract. The only solution to get sight back is through surgery, which unfortunately is prohibitively expensive to many poor people [4-6] in developing countries. Sound management of senile cataract depends upon early detection, close monitoring, and timely surgical intervention.

Auto-immune phenomena are thought to play a significant role in the initiation and propagation of several eye diseases. However, there is little evidence so far to incriminate immunological mechanisms in the pathogenesis of cataract in humans [7-10]. Lens crystallins are an example of immunologically sequestered proteins with high organ specificity and low species specificity [11]. During the past decade, the concept of the high organ specificity and auto-immunogenicity of these proteins has changed as subunits of α-crystallins were detected in the rat heart [12], skeletal muscle [13], and central nervous system [14]. These proteins also resemble small heat shock proteins [15]. The human cataractous or normal lens has been used as source of antigenic materials for investigations on circulating immunoglobulins with specificity for anti-lens crystallins [7,8,10]. These developments have provided a strong possibility of autoantibodies to lens proteins being of etiological significance in the pathogenesis of cataract.

The Maillard reaction in cataract formation has also been extensively studied in both the aged and diabetic lens. Advanced glycation end products (AGEs) of various derivations and molecular structures have been shown to be markedly elevated [16-19]. A significant increase in the concentration of AGE-like fluorophores in the aging human lens was reported in our earlier work [20]. A major change was observed during the age of 40–50 years with respect to the formation of AGE-like fluorophores [20]. Anti-crystallin autoantibodies have often been demonstrated in the serum of healthy persons, especially in patients with cataract [8]. To date, polyclonal and monoclonal anti-AGE antibodies have been found to recognize various AGEs such as carboxymethyl lysine (CML), pentosidine, pyraline, crossaline, argpyrimidine, and imidazolone as the major epitopes in cataractous lens [21-25]. Recently, for the first time, we have reported an immunochemical method for the detection and quantification of glycated lens crystallins and their circulating antibodies in diabetic rats [26]. No biochemical studies are available in the contemporary literature to identify the specific glycated lens crystallins against their specific antibodies in a broader spectrum. In continuation of this work, the present study was undertaken to develop an enzyme linked immunosorbent assay (ELISA) method for early detection of human glycated lens crystallins and their circulating antibodies during aging and cataractogenesis. Since human lens crystallins are prone to posttranslational modifications, which increase with aging [27], lens samples from donors of different age groups were used for immunochemical detection of glycated lens crystallins.

The prediction of the diseases has been correlated with the level of antibodies, detection of which could foretell early prognosis of the diseases [28]. To explore the feasibility of using serum assays for lens proteins and to gain information on the physiologic status of the lens during cataractogenesis with respect to age, an enzyme immunoassay was developed to detect the high molecular weight fraction (HMW)+α-glycated, β-glycated, and γ-glycated crystallins and their circulating autoantibodies (IgG) in sera of cataract patients during aging.

## Methods

### Materials

Bovine serum albumin (BSA-fatty acid free, RIA grade), Freund’s complete adjuvant (FCA), Freund’s incomplete adjuvant (FIA), fish gelatin, hemoglobin, anti-rabbit immunoglobulin G (IgG) labeled with horseradish peroxidase raised in goat (whole molecule), anti-human IgG labeled with horseradish peroxidase raised in goat (whole molecule), anti-rabbit IgG labeled with alkaline phosphatase raised in goat (whole molecule), 3, 3′, 5, 5′ tetramethylbenzidine (TMB), urea hydrogen peroxide, β-cyclodextrin, *p*-nitro-blue tetrazolium chloride (NBT), 5-bromo-4-chloro-3-indolyl phosphate toluidine (BCIP), Immobilon-P transfer membrane, 3,3′diaminobenzidine (DAB), and Ultrafree-MC filters (molecular weight cut-off limit–10 kDa) were all purchased from Sigma (St. Louis, MO). Sephacryl-S-200 was sourced from Pharmacia (Upsala, Sweden). Polystyrene microtiter ELISA plates were sourced from Greiner (Nurtingen, Germany). Other reagents used were of analytical grade.

### Biological materials

Human lenses (20–80 years old) were obtained from the Ramayamma International Eye Bank (a member of the International Federation of Eye Banks and Eye Bank Association of India), L V Prasad Eye Institute in Hyderabad, India. This study was undertaken with the approval of the ethical committee of L V Prasad Eye Institute. Informed consent was also obtained for collecting the cadaver lens through the Eye Bank of L V Prasad Eye Institute. The lenses were stored at –80 °C until further use.

Human sera were obtained from cataract patients (n=60, age: 40–80 years) and apparently normal subjects (n=30, age: 40–80 years) with their written consent. This study was also undertaken with the approval of the ethical committee of Sadhuram Eye Hospital in Hyderabad, India. Five milliliters (ml) of blood was obtained from each subject. The serum was separated after centrifugation and stored at −20 °C until further use.

### Preparation of human lens homogenate

Aging human lenses (20–80 years) were homogenized individually in 20 mM phosphate buffer (pH 7.4) and centrifuged at 10,000x g for 30 min at 4 °C. Each sample was separated into a clear supernatant and precipitate. The supernatant is referred to as the “soluble fraction” and the precipitate as the “insoluble fraction.” The precipitate obtained was further solubilized in 200 μl of 0.1 N NaOH. Both fractions were used for ELISA (enzyme linked immunosorbent assay) and western blot studies. Protein was estimated by the method of Lowry et al. [29].

### Isolation and synthesis of antigens

Cadaver human lenses (n=4; age: 20–30 years) were used for the isolation of crystallins (HMW+α, β, and γ) using Sephacryl-S-200 gel chromatography (113×1.5 cm, id) [30]. Rat β- and γ-crystallins were isolated by the method described earlier [31]. Glycated proteins were synthesized in vitro as described previously [32]. Briefly, proteins (BSA, hemoglobin, and human HMW+α-, β-, rat β-, and γ-crystallins; 0.1 g) were dissolved separately along with glucose (1.6 mmol) and ribose (0.7 mmol) in 5.0 ml of 400 mM sterile sodium phosphate buffer (pH 7.4). The samples were processed under sterile conditions using a laminar flow cabinet. The vials were sealed and placed in an air-circulating incubator at 37 °C for 10 weeks. After incubation, each mixture was dialyzed extensively and concentrated using a spin column (Ultrafree-MC filters, molecular weight cut-off limit-10 kDa; Sigma, St. Louis, MO). The degree of glycation was assessed by the trinitrobenzene sulfonic acid method (TNBS) [33] using the following formula:

% Conjugation=(Conc. of ε –amino group in BSA) − (Conc. of ε –amino group in BSA after glycation)/(Conc. of ε –amino group in BSA)

The synthetic AGEs were prepared in vitro as reported earlier [20]. Briefly, a few amino acids, which are present in the sequence for human γ-crystallins, were selected because of their high susceptibility to glycation/AGE formation in the course of cataract development. The following amino acid mixture was incubated: 9.5 μmoles of lysine, 100 μmoles of arginine, 23 μmoles of histidine, and 67 μmoles of tyrosine along with 1.6 mmoles of glucose, 0.7 mmoles of ribose, all in 5.0 ml of 400 mM phosphate buffer, pH 7.4. The samples were processed under sterile conditions using the laminar flow hood. The vials were sealed and placed in an air-circulating incubator at 37 °C for 10 weeks. After incubation, the mixture was dialyzed extensively and concentrated using a spin column (Ultrafree-MC filters, molecular weight cut-off limit-10 kDa).

### Production and characterization of polyclonal antibodies to human total lens proteins

Polyclonal antibodies were raised against human total lens proteins (40–80 years) in two rabbits [34]. Homogenates (10%) of the lyophilized lenses from 40, 60, and 80 years were mixed in the proportion of 1:2:1 (120 µg [40 years] + 240 µg [60 years] +120 µg [80 years]) in phosphate buffer (20 mM, pH 7.4) containing sodium chloride (100 mM) and EDTA (25 mM). In this antigen mixture, the ratio of soluble:insoluble lenticular protein was maintained as 2:1. A primer dose of 475 μg of the human total lens protein per kg of body mass was given intradermally at multiple sites. It was dissolved in sterile saline and emulsified with FCA in a 1:1 ratio. Later, three booster doses were given (250–300 μg/kg body mass) intramuscularly in FIA. Blood was collected from the animals after 30 days of primer dose and 10–12 days after the booster doses. At the end of the immunization schedule, the animals were sacrificed and blood was collected by cardiac puncture. Serum was separated, lyophilized, and stored at –20 °C until further use.

The immune response of the immunogen was checked by the double diffusion Ouchterlony technique [35]. Antisera titers for pre-immune, primer, and for I, II, and III boosters were determined by checkerboard analysis using the antibody capture assay. An immunoglobulin G (IgG) fraction from rabbit antisera was obtained by affinity purification using a commercial Protein-G column as described earlier [36]. The cross-reactivity of the affinity-purified antisera (raised against human total lens proteins) were evaluated against rat β-, β-glycated, γ-, and γ-glycated crystallins and also against glycated BSA, glycated hemoglobin (used as a test-control), and synthetic AGEs by antibody capture assay. The affinity purified antisera was used at a dilution of 1:40,000 for the cross-reactivity studies. The % cross-reactivity calculations were based on the following equation:

([Concentration of standard required to produce 50% inhibition in the immunoassay]/[Concentration of cross reacting material {antigens} required to produce 50% inhibition in the immunoassay]) x 100

### Non-competitive ELISA

The affinity-purified polyclonal antibodies that were specific to human total lens proteins were used to determine the levels of glycated crystallins (HMW+α, β, and γ) in lens by non-competitive ELISA as described in detail previously with slight modification [37]. Briefly, a 96 well microtiter plate was coated with their respective concentrations of purified antigens (human HMW+α-glycated or β-glycated, each 500 ng/50 μl per well; rat γ-glycated, 1000 ng/50 μl per well) dissolved in 100 mM carbonate buffer, pH 9.6, incubated overnight at 37 °C, and washed three times with washing buffer (phosphate buffered saline, 10 mM, pH 7.2 with 0.05% Tween-20 and 0.01% sodium azide, PBS-T). The wells were blocked for non-specific binding with 100 μl per well of 0.1% fish gelatin in PBS (10 mM, pH 7.2) and washed three times. To each well, 50 μl of diluted (1:40,000) affinity-purified polyclonal antibodies to human total lens proteins were added, which had been pre-incubated with test (human sera)/standard (different concentrations [1–1000 ng] of the human HMW+α-glycated or β-glycated or rat γ-glycated crystallins separately as antigens) for 1 h at room temperature before adding to the plate. After 1 h of incubation on the plate, wells were washed three times and later incubated at 37 °C for 1 h with 50 μl/well of diluted (in 0.5% fish gelatin in PBS) horseradish peroxidase labeled anti-rabbit IgG (1:6,000) raised in goat. The plates were washed three times, and 150 μl per well of substrate buffer was added, which consisted of 450 μl of TMB (10 mg/ml of dimethyl sulfoxide) in 15 ml of 100 mM acetate buffer (pH 5.0) containing 0.25% w/v (i.e., 37.5 mg) β-cyclodextrin and 0.015% w/v (i.e., 2.25 mg) of urea hydrogen peroxide. The reaction was terminated after 10 min by adding 50 μl per well of 1.25 M H_2_SO_4_. The absorbance at 450 nm was recorded using a SLT Spectra ELISA reader (SLT Lab Instruments, Salzburg, Austria). Percent binding of antibodies versus concentration of analyte (1–1000 ng) was used to generate an inhibition plot using linear regression analysis. The concentration of glycated crystallins (HMW+α, β, and γ) in serum was expressed as µg/ml of serum. The concentration of these immunogens is the amount of protein (HMW+α-glycated, β-glycated, and γ-glycated crystallins) required to inhibit 50% (IC_50_) of the antibody binding.

Both glycated and non-glycated crystallins (HMW+α, β, and γ) were analyzed by non-competitive ELISA where the antigens (human HMW+α-, HMW+α-glycated, β-, β-glycated, and rat γ- and γ-glycated crystallins) were used as analytes to displace the antibody. This study was performed to establish the 50% inhibition (IC_50_) level of the respective parent antigens.

### Antibody capture assay

The concentration of circulating antibodies to HMW+α-glycated, β-glycated, and γ-glycated crystallins in serum from cataract patients were analyzed by antibody capture assay as described previously [32] using the human HMW+α-glycated and β-glycated crystallins and rat γ-glycated crystallins as antigens. The microtiter plate was coated with their respective concentration of antigens (human HMW+α-glycated or β-glycated crystallin, each 500 ng/50 μl per well; rat γ-glycated, 1000 ng/ 50 μl per well) dissolved in 100 mM carbonate buffer (pH 9.6) incubated overnight at 37 °C and washed three times with washing buffer. The wells were blocked for non-specific binding with 100 μl per well of 0.1% fish gelatin in PBS (10 mM, pH 7.2). The plates were washed three times, and sera (1:5,000) from normal and cataract patients was added and incubated for 2 h at 37 °C. After washing, plates were incubated at 37 °C for 1 h with 50 μl per well of diluted (0.5% fish gelatin in PBS) horseradish peroxidase labeled anti-human IgG raised in goat (1:12,000). The plates were washed three times, and 150 μl per well of substrate buffer was added, which consisted of 450 μl of TMB (10 mg/ml of dimethyl sulfoxide) in 15 ml of 100 mM acetate buffer (pH 5.0) containing 0.25% w/v (i.e., 37.5 mg) β-cyclodextrin and 0.015% w/v (i.e., 2.25 mg) of urea hydrogen peroxide was added to each well. The reaction was terminated after 10 min by adding 50 μl of 1.25 M H_2_SO_4_ to each well. The absorbance at 450 nm was recorded using a SLT Spectra ELISA reader (SLT Lab Instruments). Affinity purified antisera to human total lens proteins (1–1,000 ng) were used to generate the calibration plot for crystallin-specific antibodies (HMW+α-glycated, β-glycated, and γ-glycated crystallins). Results were expressed as µg/ml of serum.

### Validation of ELISA methods

The ELISA methods developed were validated by using serum samples obtained from cataract patients (n=60, age: 40–80 years) and apparently normal subjects (n=30, age: 40–80 years).

### Assessment of cataractogenesis

Assessment of cataractogenesis with respect to glycation of lens crystallins in aging human lens using affinity purified antisera to human total lens proteins as primary antibody was performed by non-competitive ELISA, western blot, and immunohistochemistry.

#### Non-competitive ELISA

Non-competitive ELISA was performed both in soluble and insoluble fractions. The method has been described as detailed above.

#### Western blot

Western blot analysis in aging human lens fractions (soluble and insoluble, separately) was performed according to Towbin’s method [38] with slight modification. This was performed using 50 μg lens protein (both fractions separately) from different age groups (30–80 years). It was done to check the degree of glycation during the progress of senile cataract in soluble and insoluble fractions of lens proteins. Briefly, proteins separated by 12.5% SDS–PAGE were electrophoretically transferred to Immobilon-P transfer membrane (polyvinylidene fluoride membrane) using a CONSORT electro-blotter system (Consort N.V., Turnhout, Belgium). The membrane was soaked in 100% methanol for 30 s and washed with water for 2 min. It was then soaked in Towbin’s transfer buffer (25 mM Tris, 192 mM glycine, and 20% methanol) for 30 min. The filter papers for sandwiching the membrane and gel were cut to the dimensions of the gel and were also soaked in Towbin’s transfer buffer for 30 min. The transfer was performed at 1.1 mA/cm^2^ of gel, 50 V, and 150 W for 2 h at 21 °C. The membrane was washed three times with washing buffer (10 mM PBS with 0.05% Tween-20) and incubated in blocking buffer (3% fish gelatin in 10 mM PBS) for 30 min and washed with washing buffer. It was incubated overnight with blocking buffer containing diluted polyclonal antibodies to human total lens proteins (1:1,000) with gentle shaking at 21 °C. The membrane was washed three times with washing buffer to remove unbound antibodies and incubated for 1 h in alkaline phosphatase assay buffer (100 mM NaCl, 5 mM MgCl_2_, 100 mM Tris) containing diluted goat anti-rabbit IgG coupled with alkaline phosphatase (1:5,000). The membrane was washed three times with washing buffer and incubated with BCIP/NBT developing solution (115 mmoles of BCIP and 61 mmoles of NBT) until purple bands appeared. The color development was arrested by immersing the membrane in water. The membrane was air dried for 20 min and stored. It was photographed using a digital image documentation system (UVItec, Cambridge, England).

#### Immunohistochemistry

Immunohistochemistry study of the human lens was done with slight modification as described earlier [25] using antibodies to human total lens proteins as the primary antibody. Briefly, formalin-fixed and paraffin-embedded human cadaver lens (40–80 years) were cut into 3.0 µm thick sections and mounted on frost-free slides. After overnight drying, the sections were kept in an oven at 56 °C for 25 min, deparaffinized with xylene, and rehydrated in 95% of ethanol. To increase the immunoreactivity of the epitopes, the sections were irradiated by microwave in 10 mM sodium citrate buffer (pH 6.0) for 5 min at 800 W three times. After washing with PBS-T (pH 7.4) for 15 min, the sections were treated with 0.3% H_2_O_2_ for 15 min followed by thorough washing with PBS-T. Blocking for non-specific binding was done using 0.1% fish gelatin in PBS (10 mM, pH 7.2) with normal rabbit serum for 30 min and washed for 15 min. All steps were done at room temperature (25 °C) in a humid chamber. Sections were incubated overnight with polyclonal antibodies (1:1,000) to human total lens proteins in a humid chamber at 4 °C. Controls were maintained by replacing just the primary antibodies and both primary and secondary antibodies with PBS to determine non-specific binding of the secondary antibodies and inhibition of endogenous tissue peroxidase, respectively. After washing several times with PBS-T, sections were incubated with secondary antibodies (1:2,000), horseradish peroxidase labeled anti-rabbit IgG raised in goat, at 25 °C for 1 h in humid chamber. After incubation with the secondary antibodies, peroxidase activity was detected with DAB substrate solution (0.02% diaminobenzidine, 0.005% H_2_O_2_, 10 mM Tris-HCL, pH 7.5). Sections were incubated with DAB substrate solution for 30 min in a dark chamber at 25 °C followed by vigorous washing with distilled water. Sections were counter-stained with Mayer’s hematoxylin followed by washing with running tap water. Stained sections were permanently mounted and observed under compound microscope (Leica DM LB2; Leica Microsystems, Wetzlar, Germany), and photographs were obtained using a digital camera.

### Statistical analysis

The data was statistically analyzed by using Sigma-Stat software version 5.0 (Sigma). The test of significance was based on two-tailed Student’s *t*-test (p<0.05 and/or p<0.01).

## Results

### Degree of glycation

The degree of glycation of crystallins (human HMW+α- and β-crystallins) monitored by TNBS assay was found to be 86% at the end of 12 weeks of incubation.

### Characterization of antiserum

**Figure 1:**
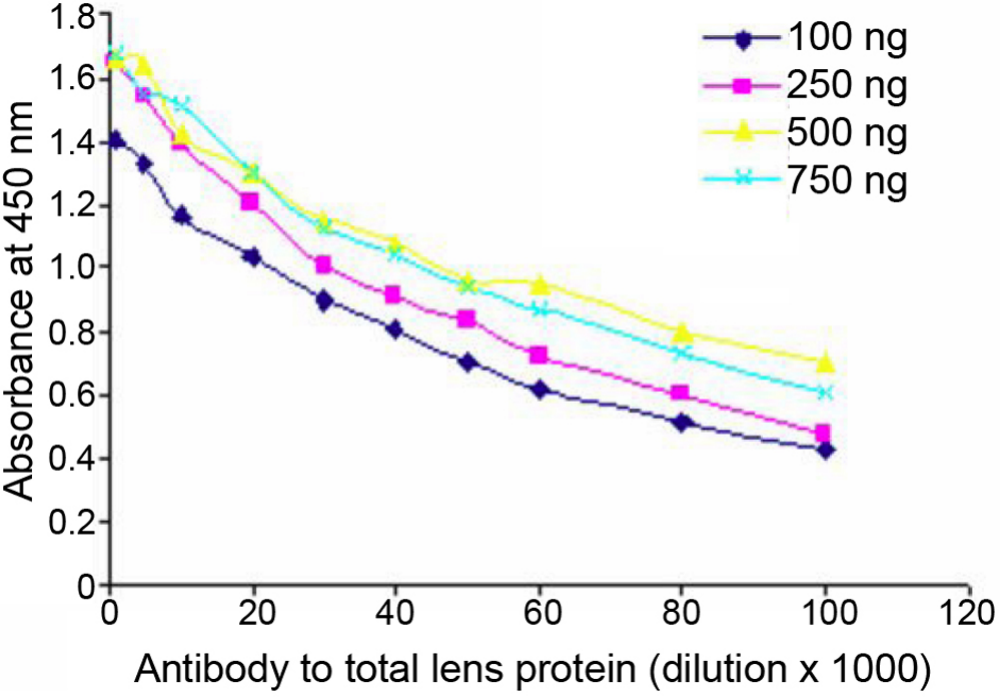
Titer determination curve for antibodies to human total lens proteins by antibody capture assay. The affinity purified polyclonal antibodies were diluted from 1:1,000 to 1:1,00,000 with a coating parent antigen (human total lens proteins) concentration of 100, 250, 500, and 750 ng. The optimal dilution obtained was 1:40,000 with 250 ng of immobilized antigen, which gave an absorbance of 0.92. Data points are mean values (n=5).

Human total lens proteins (40–80 years) were used to produce polyclonal antibodies in rabbits. The animals showed a better response, and the antiserum titer of the second booster showed better response at the dilution of 1:16 against human total lens proteins (40–80 years) as determined by the Ouchterlony double diffusion technique. Affinity-purified antibodies to human total lens proteins (40–80 years) as determined by antibody capture assay showed a titer of 1:40,000 at 250 ng per well of human total lens proteins, which gave an absorbance of 0.92 (after appropriate blank correction) (Figure 1). The higher dilution of anti-sera (1:40,000) indicates better specificity of the antibodies to the respective antigen (human total lens proteins).

**Figure 2:**
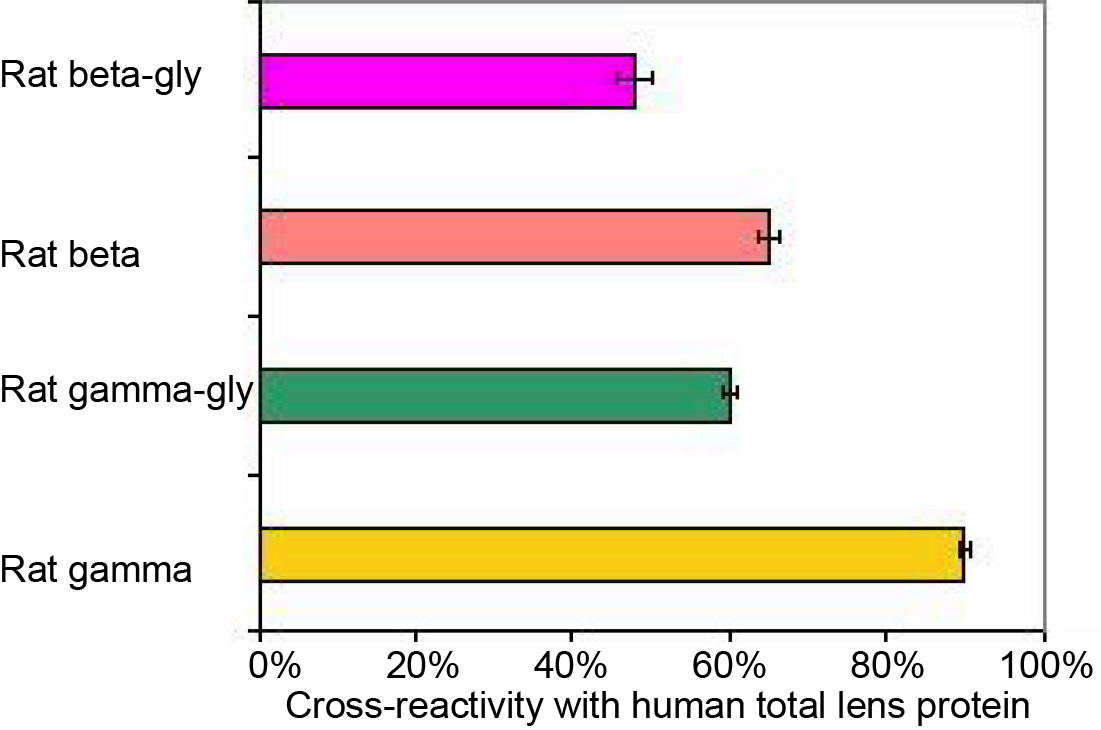
Cross reactivity of the affinity purified antisera raised against human total lens proteins with rat β, β-glycated, γ-, and γ-glycated crystallins, as determined by antibody capture assay. The affinity purified antisera was used at a dilution of 1:40, 000. Data points are mean values (n = 5).

**Figure 3:**
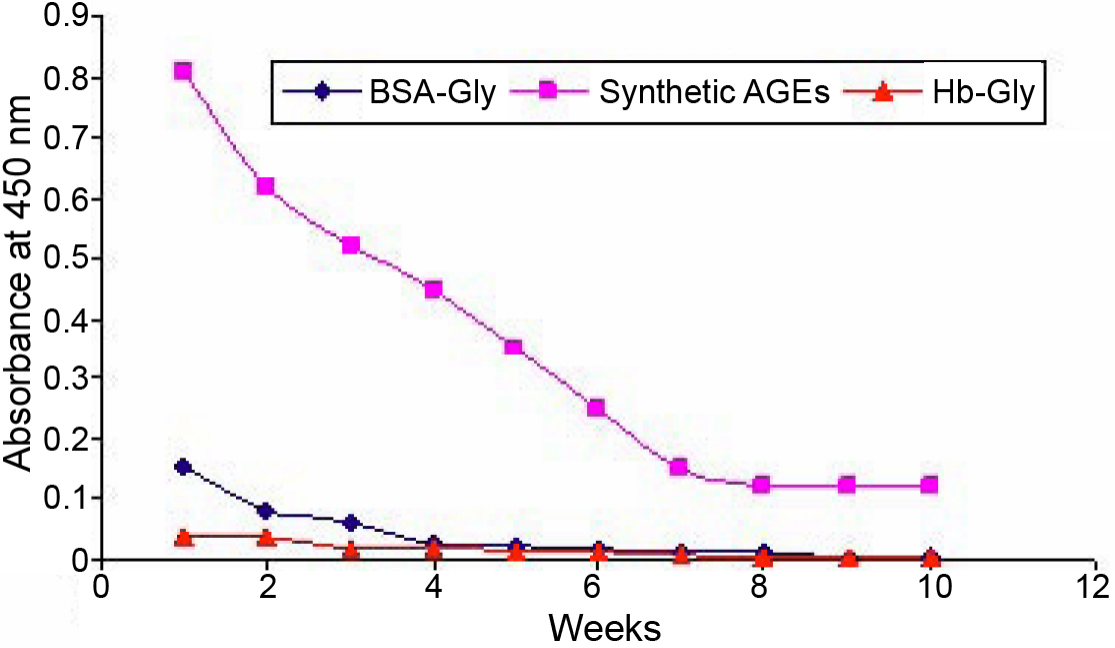
Cross reactivity of the affinity purified antisera raised against human total lens proteins with BSA-glycated, hemoglobin-glycated, and synthetic AGEs, as determined by antibody capture assay. The affinity purified antisera was used at a dilution of 1:40, 000. Data points are mean ± SD (n = 5).

Antibodies to human total lens proteins (age 40–80 years) showed cross-reactivity with rat β- (65%), β-glycated (45%), γ- (90%), and γ-glycated (60%) crystallins as shown in Figure 2. These antibodies did not show any cross-reactivity with glycated BSA and glycated hemoglobin but have shown immune response to the early week (I and II) products of chemically synthesized AGEs as shown in Figure 3.

### Non-competitive ELISA

**Figure 4:**
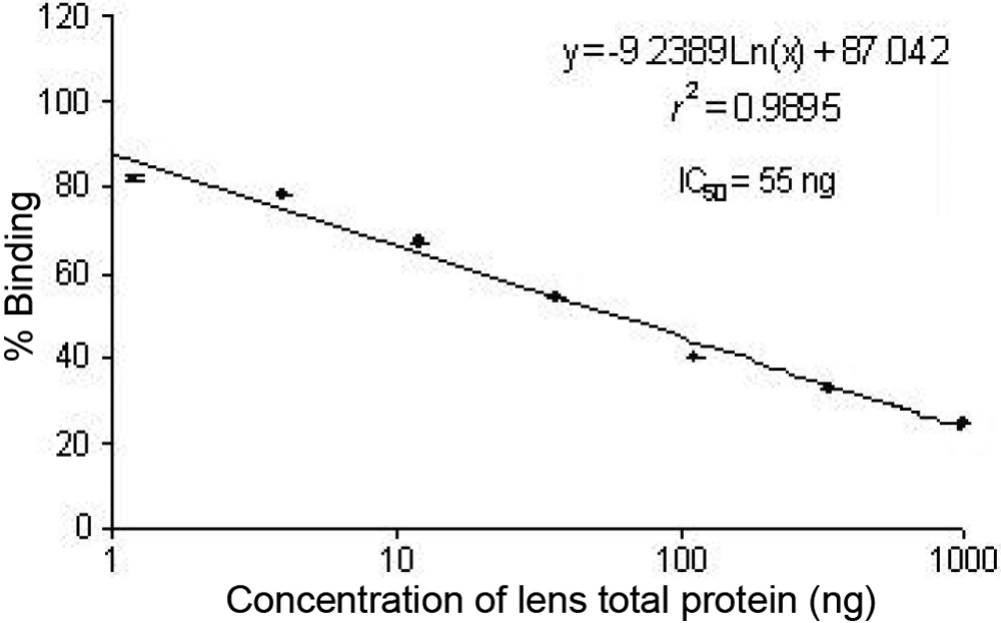
Standard displacement plot for human total lens proteins as determined by non-competitive ELISA. Percent binding of antibodies versus concentration of human total lens proteins (1-1,000 ng) was used to generate an inhibition plot using linear regression analysis. The affinity purified antisera was used at a dilution of 1:40,000. Data points are mean ± SD (n = 5).

The standard displacement curve for various concentrations (1–1,000 ng) of human total lens proteins is shown in Figure 4. The inhibitory concentration at 50% antibody binding (IC_50_) for this protein was found to be 55 ng based on regression analysis, r^2^=0.99. The immune response of antisera to human total lens proteins (40–80 years) with both glycated and non-glycated crystallins (HMW+α, β, and γ) was also calculated based on regression analysis, which is summarized in Table 1. Further, it was noted that the IC_50_ values for the lenticular crystallins were less than that of their respective glycated crystallins. Among the native crystallins (HMW+α, β, and γ), the IC_50_ values for HMW+α-crystallin (16.45 ng) were found to be significantly lower as depicted in Table 1.

**Table 1:** The IC_50_ values of various antigens as determined by non-competitive ELISA using affinity purified IgG fraction from the antisera raised against human total lens proteins.

**Antigens (Ag)**	**IC_50_ (based on regression analysis)**	**r^2^ value**
Human total lens protein	55 ng	0.99
Human HMW+α	16.45 ng	0.99
Human HMW+α-gly	273 ng	0.95
Human β	37.82 ng	0.99
Human β-gly	260 ng	0.91
Rat γ	105.34 ng	0.98
Rat γ-gly	313 ng	0.97

Both glycated and non-glycated lens crystallins were used for the analysis. The affinity purified IgG fraction was used at a dilution of 1:40, 000. The regression coefficients (r^2^) indicate the linearity of the displacement curve.

### Validation of ELISA

**Figure 5:**
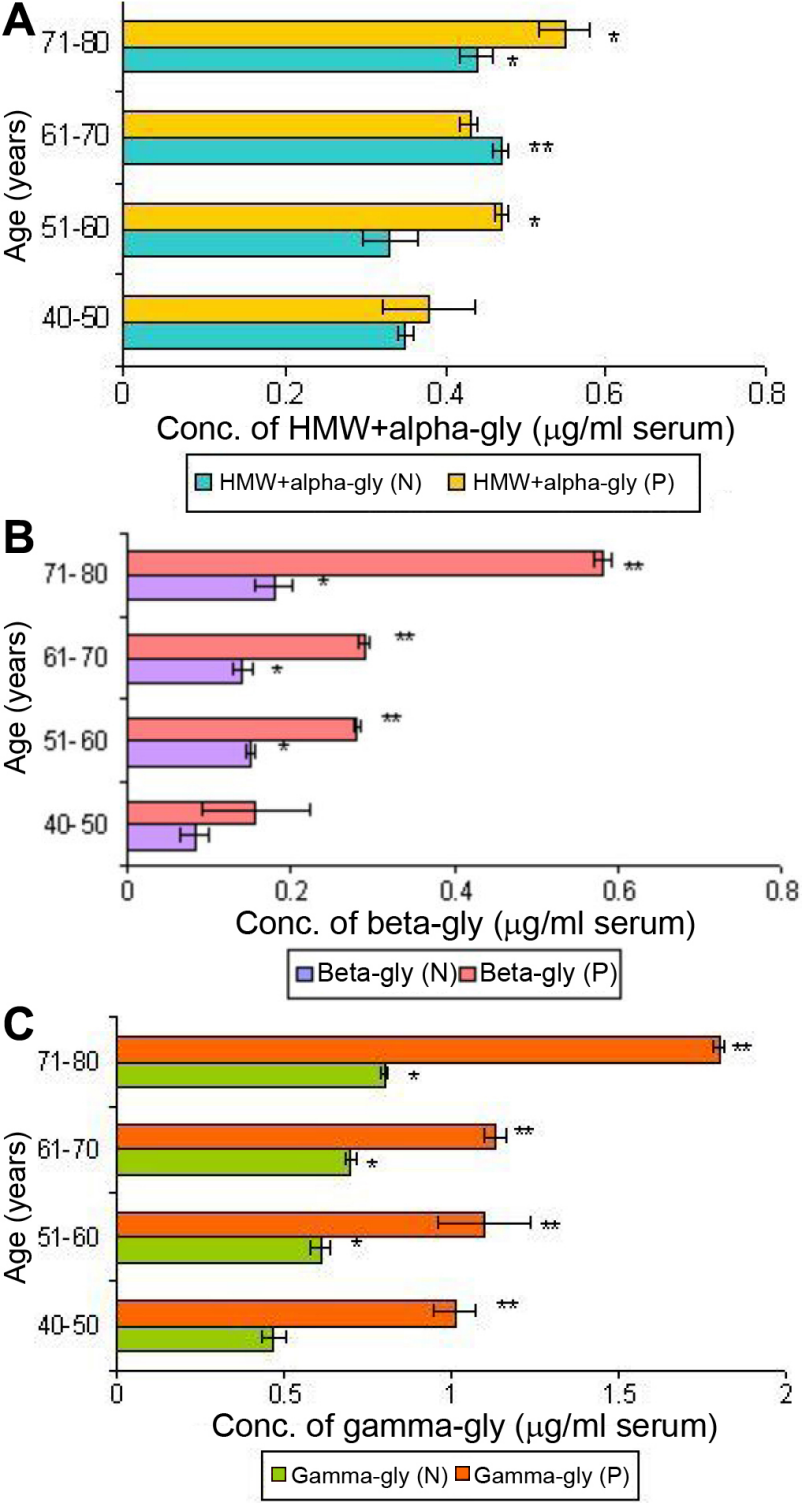
Profile of glycated human lens crystallins for all age groups in peripheral circulation. **A:** Profile of HMW+α-glycated crystallins in peripheral circulation during aging with respect to cataractogenesis as determined by non-competitive ELISA using polyclonal antibodies raised against human total lens proteins. Values are mean±SD at every given time point (n=5). The asterisk indicates that p<0.05, and the double asterisk indicates that p<0.001. N-apparently normal subjects; P-cataract patients. **B:** Profile of β-glycated crystallins in peripheral circulation during aging with respect to cataractogenesis as determined by non-competitive ELISA using polyclonal antibodies raised against human total lens proteins. Values are mean±SD at every given time point (n=5). The asterisk indicates that p<0.05, and the double asterisk indicates that p<0.001. N-apparently normal subjects; P-cataract patients. **C**: Profile of γ-glycated crystallins in peripheral circulation during aging with respect to cataractogenesis as determined by non-competitive ELISA using polyclonal antibodies raised against human total lens proteins. Values are mean±SD at every given time point (n=5). The asterisk indicates that p<0.05, and the double asterisk indicates that p<0.001. N-apparently normal subjects; P-cataract patients.

Circulating glycated crystallins (HMW+α, β, and γ) were observed in sera of both apparently normal subjects and cataract patients from different age groups (40–80 years) as shown in Figure 5A-C. There was a statistically significant change (p<0.001) in the levels of circulating β-glycated and γ-glycated crystallins in the age group of 40–80 years with their respective controls. This was not true for the HMW+α-glycated crystallins. Notably, the levels of serum γ-glycated crystallins were found to be threefold higher than that of HMW+α-glycated and β-glycated crystallins in the age group of 70–80 years. Age matched controls also showed significant change (p<0.05) in the levels of glycated crystallins during aging.

**Figure 6:**
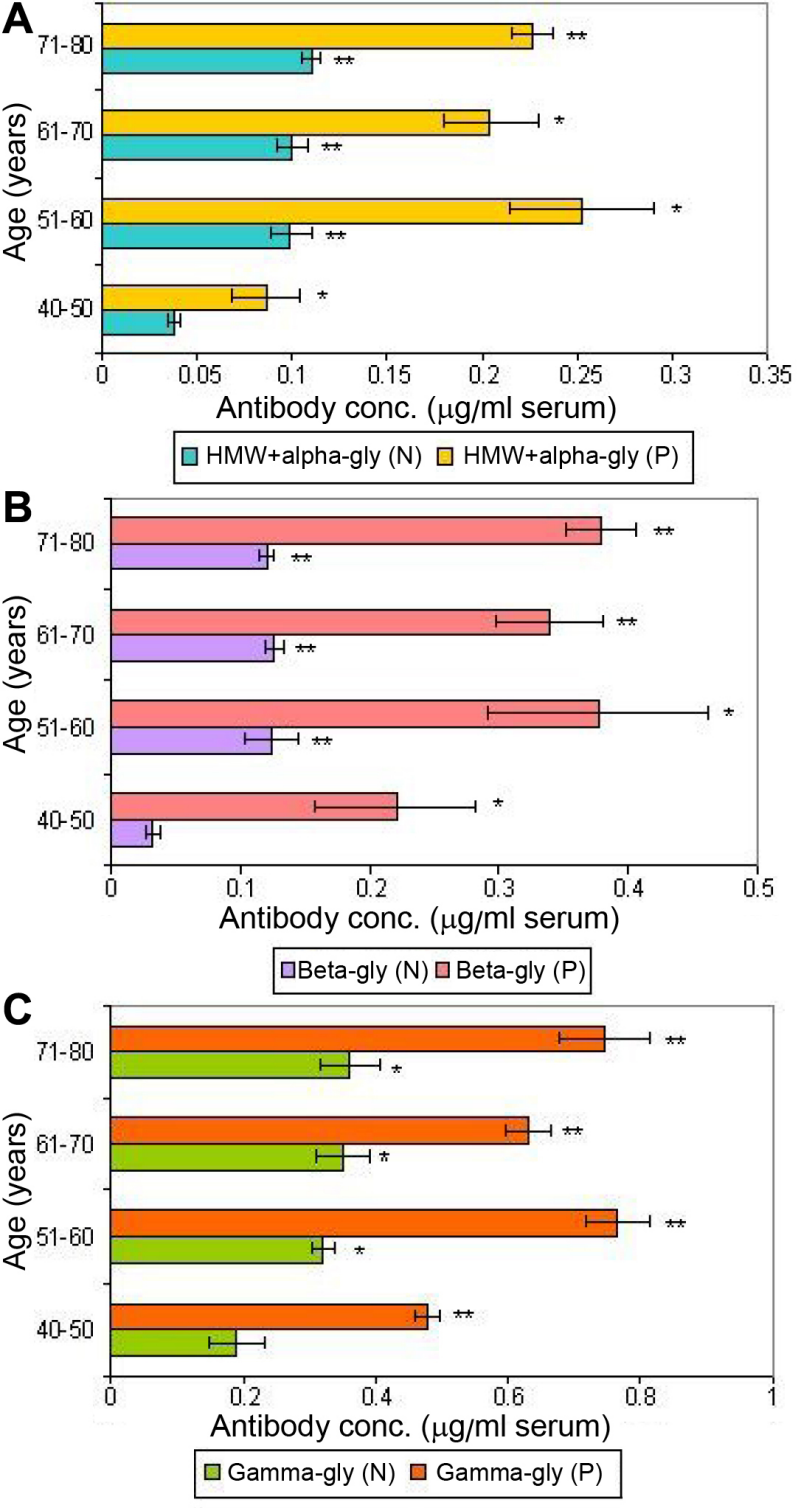
Profile of circulating autoantibodies to glycated crystallins in human serum. **A:** Profile of circulating autoantibodies to HMW+α-glycated crystallins in serum during aging with respect to cataractogenesis as determined by antibody capture assay using polyclonal antibodies raised against human total lens proteins. Values are mean±SD at every given time point (n=5). The asterisk indicates that p<0.05, and the double asterisk indicates that p<0.001. N-apparently normal subjects; P-cataract patients. **B:** Profile of circulating autoantibodies to β-glycated crystallins in serum during aging with respect to cataractogenesis as determined by antibody capture assay using polyclonal antibodies raised against human total lens proteins. Values are mean±SD at every given time point (n=5). The asterisk indicates that p<0.05, and the double asterisk indicates that p<0.001. N-apparently normal subjects; P-cataract patients. **C**: Profile of circulating autoantibodies to γ-glycated crystallins in serum during aging with respect to cataractogenesis as determined by antibody capture assay using polyclonal antibodies raised against human total lens proteins. Values are mean±SD at every given time point (n=5). The asterisk indicates that p<0.05, and the double asterisk indicates that p<0.001. N-apparently normal subjects; P-cataract patients.

Circulating autoantibodies to glycated crystallins (HMW+α, β, and γ) were detected in the serum of apparently normal subjects and cataract patients in the age group of 40–50 years as given in Figure 6A-C. The levels of these autoantibodies were significantly higher (p<0.05 and p< 0.001) at every time point with their respective controls. Autoantibodies to γ-glycated crystallins (Figure 6C) were found to be twofold and 3.2 fold higher as compared to the levels of autoantibodies to β-glycated (Figure 6B) and HMW+α-glycated (Figure 6A) crystallins, respectively.

### Assessment of cataractogenesis

**Figure 7:**
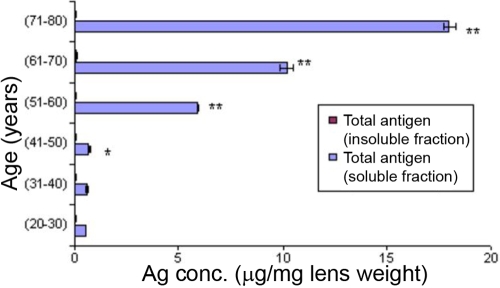
Immunochemical characterization of antibodies to human total lens proteins towards the soluble and insoluble proteins of aging human lens protein by non-competitive ELISA. Values are mean±SD at every given time point (n=5). The asterisk indicates that p<0.05, and the double asterisk indicates that p<0.001 in comparison to 20–30-year-old lens fractions.

**Figure 8:**
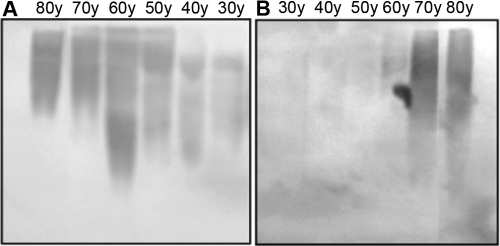
Western blot analysis of antibodies to human total lens proteins for all age groups towards the soluble and insoluble protein fractions of aging human lens. Lens protein (50 μg) from (**A**) soluble and (**B**) insoluble fractions was loaded and were separated by SDS-PAGE (12.5%). Affinity purified antibodies were used at a dilution of 1:1,000.

**Figure 9:**
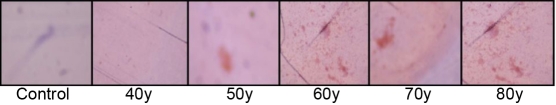
Immunohistochemical detection of glycated crystallins in aging human lens from 40-80 years using affinity purified antibodies raised against human total lens proteins. The control includes the replacement of primary antibodies by PBS. Human cadaver lens (40–80 years) were cut into 3.0 µm thick sections. Affinity purified antibodies were used at a dilution of 1:1,000. All photographs were taken using 40X objective.

Apparent changes (p<0.05) in the degree of glycation were found in the age group of 41–50 years as compared to the age group of 20–40 years by the ELISA methodology (Figure 7). A significant change (p<0.001) was observed from 51 to 60 years in the soluble fraction of the lens while the insoluble fraction of the lens did not show any change with respect to the degree of glycation. Western blot analysis (Figure 8) illustrates a similar pattern of glycation in the soluble fraction (Figure 8A) of aging human lens as observed by ELISA. A typical trend of glycation was not observed in the insoluble fraction (Figure 8B) of aging human lens. Older lens (70–80 years) did show a comparative degree of glycation in the insoluble fraction of human lens as compared to the younger lens (30–50 years; Figure 8B). Immunohistochemical analysis indicated enhanced immunostaining (intensity) in the aged (60–80 years) lens compare to 40-year-old lens. Control sections did not show any immunostaining (Figure 9).

## Discussion

Considerable changes have been observed in aging human lens with respect to opacity and coloration during the age of 40–50 years with respect to the formation of AGE-like fluorophores as reported earlier [26]. Hence, selection of human lenses from the age group of 40–80 years was done to raise polyclonal antibodies to human total lens proteins that carry a varying degree of glycation during the natural course of aging.

Higher specificity of these antibodies to human total lens proteins (1:40,000) was observed as these comprise the major lenticular structural proteins (α-, β-, and γ-crystallin). The unique combination of lens crystallins sourced as the immunogen from the 40–80 years age group possibly resulted in high titer polyclonal antibodies capable of detecting the changing profile of crystallins (HMW+α, β, and γ) during the course of aging. Production of polyclonal antibodies to human total lens proteins resulted in antibodies that were capable of cross-reacting with synthetically prepared rat glycated crystallins (β and γ) and AGEs. The higher percent of cross-reactivity (90%) between rat γ- crystallins and antibodies to human total lens proteins indicates that both share common antigenic determinants. Based on the results of this cross-reactivity experiment, rat γ-crystallins could be used successfully as a substitute for human γ-crystallins. Human hemoglobin and albumin are among the many proteins that undergo non-enzymatic glycation (degree of glycation ~7%–12%) [39,40]. Their levels of glycation are found to be elevated during the diabetic condition [41]. Therefore, it was necessary to rule out the possible cross-reactivity of these polyclonal antibodies with glycated BSA and hemoglobin before using these antibodies for the detection of lenticular glycated proteins. The polyclonal antibodies to human total lens proteins did not show any cross-reactivity with synthetically prepared glycated BSA and glycated hemoglobin (Figure 3).

The vivid range of specificity of these polyclonal antibodies has been supported by the IC_50_ values of glycated and native crystallins (HMW+α, β, and γ; Table 1). This indicated lower specificity towards glycated proteins as compared to their native form in all cases since the major epitopes possibly get masked due to glycation. The higher sensitivity of antibodies to HMW+α-crystallins (IC_50_=16.45 ng) and lesser sensitivity to γ-crystallins (IC_50_=105.34 ng) supports the evidence that older lens and cataract lens have decreased levels of γ-crystallins in the soluble fraction and increased levels of HMW+α-crystallins [42,43].

Several studies have implied that cataract is an autoimmune disorder [7,8,44-48]. In mature cataracts, longstanding leakage of crystallins through the damaged capsule is likely to occur. The immune system is thus exposed to the crystallins, which are normally sequestered in the lens. However, those crystallins that normally occur in small amounts outside the lens would be recognized as self and therefore, normally would not evoke an autoimmune response. Only the truly lens-specific crystallins would be recognized as a non-self antigen and trigger autoantibodies formation. The different types of lens-specific glycated crystallins such as HMW+α, β, and γ in human sera from cataract and apparently normal aging subjects (Figures 5A-C) as well as their respective autoantibodies in the same set of subjects (Figures 6A-C) were detected using affinity-purified polyclonal antibodies. This is the first report where the polyclonal antibodies raised against human total lens proteins have been used successfully to detect the lens-specific glycated antigens and their circulating antibodies in human.

Since human lens crystallins are prone to post-translation modifications, which increases with aging [20], the usage of lens samples from different ages (40–80 years) may have the wider epitopes for different type of AGEs. Hence, the concentration of circulating autoantibodies to glycated crystallins (HMW+α, β, and γ) at the age of 40 years and onwards was found to be significantly higher in the test samples in comparison to their age matched controls (Figures 6A-C). Further, in the age group of 50–60 years, the levels of these autoantibodies reflected maximum change. This could possibly be due to the vivid nature of polyclonal antibodies to the early stage of glycation, which reflected enhanced levels of glycated products as early as 40–50 years.

Questions concerning the role of glycation with respect to the pathology of cataractogenesis during aging have been assessed in the present study using ELISA, western blot analysis, and immunohistochemical methods (Figure7, Figure 8, and Figure 9). Both western blot and ELISA results confirmed the specificity of polyclonal antibodies, particularly in soluble fraction of lens protein, where the obvious nature of glycation starts from 41 to 50 years (Figure 7). Thus, both quantitative and qualitative evidence indicated a greater degree of glycation in the soluble fraction in comparison to the insoluble fraction of aging lenses (Figure 7 and Figure 8). This is the first report in which a greater degree of glycation in the soluble fraction of the lens has been observed when comparing to the insoluble fraction of lens by using the polyclonal antibodies that were raised against human total lens proteins. Prior reports illustrate the presence of more AGE-like products in the insoluble fraction of aging and cataract lens compared to the soluble fraction of lens [49,50] using antibodies to synthetic glycated BSA. Possibly the use of the IgG fraction of booster III anti-sera raised against naturally glycated total lens proteins are responsible for this distinctive result wherein the nature of epitopes may be different from the one generated synthetically.

It is interesting to note the obvious changes in the levels of circulating autoantibodies against HMW+α-glycated, β-glycated, and γ-glycated crystallins in the serum of cataract patients in the age group of 40–50 years. A similar trend was observed for circulating antigens and β-glycated and γ-glycated crystallins in the age group of 70–80 years in comparison to their age matched controls (Figure 5 and Figure 6). This reflects the leakage of these glycated crystallins at the age of 40–50 years in cataract patients, which is getting neutralized by their respective autoantibodies at the early age and reaching to the level of saturation by the age of 70–80 years in cataract patients. The higher level of serum γ-glycated crystallins and their autoantibodies compared to other glycated crystallins (Figure 5 and Figure 6) suggested γ-glycated crystallins to be more immunogenic than the glycated form of other crystallins, which is similar to the observation recorded for their native forms [8,32]. The γ-glycated crystallins seem to be involved in very early cataractogenic events whereas HMW+α-glycated and β-glycated crystallins may be involved in later stages. HMW+α-glycated crystallins, being an aggregated molecule as compared to β-glycated and γ-glycated, may not evoke a high level of antibody response in peripheral circulation (Figure 5 and Figure 6).

One of the major observations made in the present study is the presence of antigens and autoantibodies to glycated crystallins (HMW+α, β, and γ) in peripheral circulation in cataract patients as compared to age-matched, apparently normal subjects. γ-glycated crystallins may be considered a more suitable biomarker than β-glycated and HMW+α-glycated crystallins for the early detection of cataract during aging. However, this observation needs to be further substantiated by extending the analysis to a larger sample size of human subjects in relation to cataract development as a function of aging.

The experimental findings of the present investigation may have practical relevance in detecting the glycated crystallins (HMW+α-, β-, and γ-crystallins) and their specific autoantibodies as a biochemical marker for early detection of cataract in human subjects. Further, the immunoanalytical methods developed might have immense value in adopting appropriate strategies to delay the process of cataract formation by nutritional and metabolic interventions.
